# Animals and Cities: A Reflection on Their Potential in Innovating Nature-Based Solutions

**DOI:** 10.3390/ani14050680

**Published:** 2024-02-21

**Authors:** Giulia Granai, Carmen Borrelli, Chiara Mariti, Francesco Di Iacovo

**Affiliations:** Department of Veterinary Science, University of Pisa, Viale delle Piagge 2, 56124 Pisa, Italy; carmen.borrelli@phd.unipi.it (C.B.); chiara.mariti@unipi.it (C.M.); francesco.diiacovo@unipi.it (F.D.I.)

**Keywords:** nature-based solutions, animals, human–animal interaction

## Abstract

**Simple Summary:**

Animals have always lived with humans, but their presence in cities is growing. This phenomenon warrants a specific reflection on the advantages of human–animal interactions as a potential nature-based solution (solutions that are inspired and supported by nature, which provide environmental, social, and economic benefits). This article aims to provide an overview of the current situation of animals in cities and to explore the roles of animals and their interactions with humans in such a context. Through the lens of the European project IN-HABIT in Lucca (Italy) (which aims to codify an integrated policy on the relationship between people and animals that will then be transferred and replicated in other cities), we investigated all these aspects. In addition, our work suggests the need for the involvement of different stakeholders in the implementation of actions that aim to valorize human–animal relationships and their positive effects.

**Abstract:**

In recent decades, nature-based solutions (NBSs) have spread in scientific research, and they are increasingly deployed in cities’ strategic planning. While the number of nonhuman animals in cities is growing, a specific reflection on the advantages of human–animal interactions as potential NBSs is still lacking. This article aims to provide an overview of the current situation of animals in cities and to explore the roles of animals and their interactions with humans in such a context. These topics are crucial to the European project IN-HABIT in Lucca (Italy), which aims to codify an integrated policy on the relationship between people and animals; its outputs will then be transferred and replicated in other cities. This article concludes by highlighting the need for the involvement of different stakeholders in public–private–people partnerships to implement actions that aim to valorize human–animal relationships and their positive effects. This study presents a perspective on the relevance of animal NBSs to increase the quality of life in cities, both for citizens and for animals living in cities, and to also introduce the opportunity to develop an integrated animal urban policy able to valorize human–animal interactions in cities.

## 1. Introduction

Cities are expected to host the increasing world population in the future, generating expectations as well as questions regarding how to ensure services and future prosperity [1] for their inhabitants. The urbanization trend in society moves in parallel with growing challenges related to environmental and social aspects in addition the economic aspects. Alongside the population growth in urban contexts, social diversity and inequalities have also increased in recent decades [2,3,4,5]. Traditionally, economic opportunities have been linked to the development of the private economic sector, while it has been the role of the state to intervene and regulate access to environmental resources and ensure basic societal needs [6]. Emerging environmental and societal challenges are seen as part of a neo-liberal economic approach that disconnects value creation at the territorial level and especially access to environmental and societal public goods [7]. In times of fiscal crisis [8] and budget constraints of public administrations and municipalities [9], the provision of public goods has started to become a topic of discussion.

In addition to environmental issues, cities are also tackling emerging societal inequalities linked to their reorganization and socio-geographical fragmentation. The emerging urban inequalities regard suburban and central areas, gentrification, aging, and interethnic, intergenerational, and income divides. These societal trends are culminating in new problematic issues, such as an increasing NEET and youth learning disabilities ratio, isolation, gender inequalities, and labor market divides [10,11].

All societal needs used to be tackled by the public welfare and social/health services. Nowadays, welfare reorganization is under discussion, both in the cities and at the country level [12,13,14], and the so-called post-welfare cities [15] look to shadow care infrastructures. They emerge from the involvement of new actors and resources organized by a new mix of public institutions, NGOs, and new actors designing innovative and still unformalized practices. Such a cluster of new initiatives represents the struggle and the starting point for a transformative scenario able to redesign more inclusive infrastructures in urban areas. Such novelties might then be developed toward transition management paths [16].

In Europe and abroad, the opportunity to reshape public intervention, the involvement of local stakeholders (private and citizens), the mobilization of existing local resources, the provision of ecosystem services [17], and the promotion of the innovative use of nature are becoming bricks of an ongoing possible positive emerging scenario to improve local quality of life. In such a scenario, the so-called nature-based solutions (NBSs) are seen both as an opportunity for life improvements and as possible activators of innovative paths and partnerships among diverse public and private actors. The (co-)design of NBSs is mainly debated to tackle environmental issues (such as pollution, warming cities, and water management) but also to redesign public spaces for more positive interactions among citizens and the generation of more attractive cities (for inhabitants, investors, and visitors alike) [2,10,18,19,20].

The discussion on public goods and NBSs focuses on the possible space to introduce suitable and unconventional solutions to tackle emerging societal needs in society and how to foster their use to reduce disparities and problematic environmental impacts. At the same time, the debate on NBSs mainly focuses on green/plant-based solutions without considering the possible role of animals, although the presence of animals in cities has a historical role [21,22].

Nowadays, there is a new increasing presence in terms of numbers as well as in the role of animals in cities. In Western countries, the growing presence of animals is mainly a personal/family choice to introduce and start relationships with nonhuman animals in everyday life (dogs, cats, birds, and unconventional companion animals, but also traditionally food-producing animals). In addition, wild animals also live in cities. The wider presence of animals in cities opens space for a new reflection on their possible impacts in an urbanizing society from different perspectives. As with plants, animals might offer support to urban dwellers and generate opportunities for improving the urban quality of life as well as for supporting people in need, strengthening individual quality of life, and contributing to new societal supports [23,24].

This article begins with the growing debate on nature-based solutions in cities and their possible role in the provision of environmental, social, and economic opportunities. Then, we underline the lack of attention on the possible role of nonhuman animals in cities and the possible outcomes of their interactions with humans. Such reflection starts from the European project Horizon 2020 IN-HABIT (“INclusive Health and wellBeing In small and medium size ciTies”) in smart cities, in which the city of Lucca is nominated to become the first European human–animal city with an integrated policy on human–nonhuman animal interactions. This article focuses on the idea that the presence of animals in cities is growing as part of an individual/private nature-based solution and that cities are hosting an increasing number of nonhuman animals. At the same time, there is a lack of specific reflection on the planning of innovative, integrated policies regarding nonhuman animals’ presence in everyday life and how to take advantage of their interactions with humans. The possible outcomes emerging from innovative views on human–nonhuman animal interactions, for different circumstances and needs, are further investigated every day in terms of support for elders [25,26], children with learning difficulties, people experiencing homelessness [27,28], and people who are less empowered and isolated toward the improvement in the health of interacting humans. Meanwhile, another side of the debate looks at animals and their rights in society in terms of citizenship and human stewardship. In such a perspective, the interactions between humans and nonhuman animals could be seen as a part of a broader public discourse. This discourse could generate the foundation for a common recognition of human and nonhuman animals (both human companions and wild animals) in cities, of their common needs, and of the positive outcomes of the promotion of their interactions. Nonhuman animals can be considered as an existing *resource* (intended as beings whose presence can offer support, not as things to be exploited) that can be mobilized in innovative ways for increasing health and well-being in cities. In such respects, the topic of nonhuman animals might be part of the discussion on innovative nature-based solutions for smart and requalified urban settings, especially in times of public resource scarcity and increasing societal needs toward the organization of transition pathways able to recognize their role in the reorganization of environmental and social public goods.

This study aims to better analyze the role of animals from new and intriguing perspectives and to find solutions to overcome the lack of valorization of the presence of nonhuman animals in enhancing the quality of life of people living in urban areas. The process of social innovation to valorize the benefits derived from human–animal bonds is investigated in this article.

## 2. Nature-Based Solutions as an Evolutionary Concept

### The Current Debate on NBSs

The concept of nature-based solutions (NBSs) has become, in recent decades, more popular both in the scientific literature and in governmental and non-governmental policies following the introduction given by the European Commission (EC) and the International Union for Conservation of Nature (IUCN) [29]. The definition of NBSs given by the EC is “solutions that are inspired and supported by nature, which are cost-effective, simultaneously provide environmental, social and economic benefits and help build resilience; such solutions bring more, and more diverse, nature and natural features and processes into cities, landscapes, and seascapes, through locally adapted, resource-efficient and systemic interventions” [30]. The International Union for Conservation of Nature (IUCN) proposed a slightly different definition of NBSs as “actions to protect, sustainably manage, and restore natural or modified ecosystems, that address societal challenges effectively and adaptively, simultaneously providing human well-being and biodiversity benefits” [31].

**Figure 1:**
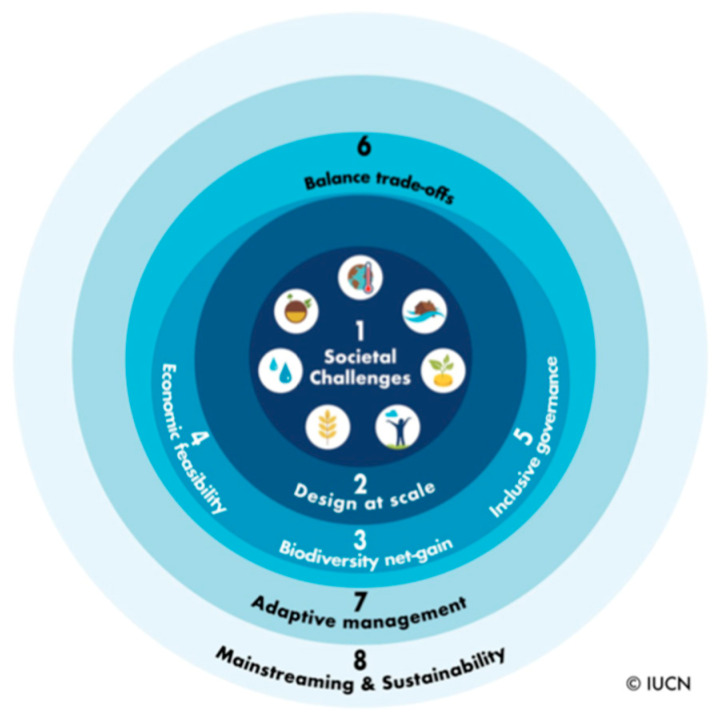
The eight criteria of the IUCN Global Standard [35].

In the literature, many studies refer to NBSs as an “umbrella” concept for different green concepts [32] that promote the role of nature in different processes, but at the same time, there is still an ongoing discussion on the extent and types of interventions that can be classified as NBSs [33]. To clarify the concept of NBSs, both the IUCN and the European Commission defined priority areas that could be addressed by NBSs, and the IUCN also suggested eight criteria by which to shape green/blue interventions as NBS actions. The IUCN defined the following five priority areas as particularly topical: water security, food security, human health, disaster risk reduction, and climate change [31]. The European Commission identified seven priority areas, most of which (NBSs for regeneration and well-being in urban areas, carbon sequestration, coastal resilience, watershed management, and ecosystem restoration) conform with the IUCN priorities. However, the European Commission also added two different and new topics—NBSs to enhance the insurance value of ecosystems and foster sustainable use of matter and energy [34]. The IUCN has drawn up a Global Standard [35] on NBSs aimed to be used as a framework for the various stakeholders—governments, planners, businesses, donors, and financial institutions—to design and support NBSs. The Standard [35] contains the following eight criteria (Figure 1): (1) address societal challenges; (2) landscape scale of intervention; (3) biodiversity gain; (4) economic viability; (5) governance capability; (6) equitably balance trade-offs; (7) adaptive management; and (8) mainstreamed within an appropriate jurisdictional context.

In the EC document on NBSs of 2021 [36], three categories depending on the degrees of intervention are proposed:

1. “Minimal or no intervention in ecosystems—or better use of protected/natural ecosystems”: interventions aimed at preserving or improving the delivery of ecosystem services by targeted ecosystems.
2. “Management approaches that involve some intervention—NBS that support sustainable, multi-functional managed ecosystems”: solutions directed toward sustainable, multi-functional ecosystems to improve them.
3. “Extensive, intrusive management of ecosystems—or the design and creation of new ones”: these solutions are focused both on the interrelation between biodiversity conservation and landscape architecture and on the integration of new approaches.

These categories and types of approaches are not full-scale solutions, and there can be a lot of different NBSs encompassing space and time.

According to the “Naturvation” project, in addition to meeting twelve societal challenges, NBSs address specific United Nations Sustainable Development Goals (SDGs). Since NBSs can lead to different benefits for society, the economy, the environment, and human well-being, among the SDGs mentioned we can also find SDG3 “Health and well-being”, which is the focus of the project we are working on.

As previously mentioned, there is great interest in the NBS theme, and this is also increasing every year. In the literature, we have found several works on this topic that refer to different kinds of interventions involving various sources related to nature.

The focus on the NBS concept originated with the aim of enhancing resilience in the context of climate change [37]; however, its potential in addressing socio-economic challenges along with environmental issues has since been increasingly recognized [34]. However, according to the review of Ershad Sarabi et al. [38], in the literature, we can mainly find NBSs with challenges in water management and enhancing water resilience (Table 1). The review also stated that significant attention has been given both to climate change adaptation and carbon sequestration and the socio-economic benefits of NBSs. It is also reported [39] how NBSs can create space for new relations and a sense of place among people in their communities, especially people who are vulnerable. This advantage is described mainly in relation to urban gardens and small-scale experiments where citizens directly participate in the management, maintenance, and monitoring of NBSs [40,41,42]. Furthermore, we can find various studies [43,44,45], where NBSs play a role in improving mental health and well-being.

Even if, as stated, the topic of NBSs has been spreading in recent years, unfortunately, the number of solutions is still low, and implementations are often slowed down by barriers in governance [37,38]. A clear assessment of the impacts of NBSs within and across different societal challenges would help in the process of recognition of their benefits by governance. In this regard, many attempts have been made to systematize the possible outcomes by introducing specific indicators [46], although the diversity and complexity of any approach make comparisons challenging.

When NBSs are implemented to use natural processes in urban contexts, they may simultaneously provide co-benefits for biodiversity and human well-being [31] but existing works only framed and evaluated benefits regarding single challenge areas (i.e., ecosystem service values, synergies, and trade-offs [47], and the co-benefits of climate interventions [48]). Additionally, there is a lack of targeted counseling for the processes that enable the consideration and assessment of co-benefits within and across the stages of implementation and decision making [49]. This leads to the fact that, as stated in different studies [50,51], the ecosystem service concept is being taken into consideration by policy, but with approaches that are too sector-based and not sufficiently sensitive to specific political and socio-cultural contexts. To overcome this gap, there is a need for more participatory processes able to amplify marginalized voices in urban co-planning, co-design, co-deployment, and co-management of NBSs. According to the topics under discussion, participatory processes should consider most stakeholders’ perceptions, including transdisciplinary working methods, co-production of knowledge and adaptive management [52], the co-creation and design of NBSs [53], as well as education and greater effort in monitoring and assessing the multiple benefits of NBSs [54]. We can also consider that some of the NBSs are linked to more infrastructural plans related to the urban rebuilding of public spaces (“hard solutions”, e.g., new squares and spaces to safely channel over raining water), while others (“soft solutions”) have a larger impact on the urban organization of an innovative dialogue and the organization of partnerships between private and public agencies toward the definition of new levels of subsidiarity and proactive participation in the everyday organization of public spaces and activities [55,56,57].

## 3. The Noisy Silence on Animal Nature-Based Solutions

Although the impact of animals on human psychological well-being is reported in many case studies [58,59,60,61,62], most of the works on NBSs usually refer to solutions that are based on the use of plants for diverse purposes [63,64], while the possible outcomes emerging from human–animal interactions are still completely underestimated. Animal NBSs refer to the possible useful role of animals as NBSs themselves, and the enhancement of human–animal relationships as a potential opportunity to increase the quality of life of urban inhabitants in cities.

Animals in cities are present in a growing number, generating everyday interactions with the urban environment and humans as well [65,66,67]. Human–animal interactions might also differ in time and specific geographic conditions due to cultural, environmental, historical, and societal arrangements. Animals might differ in species, numbers, and levels of interdependency/dependency with humans in diverse geographic settings. They live in cities, although they are always at the border of public life and experience, and their presence is taken for granted and unconsidered, other than the possible negative impacts on humans (in the case of car accidents, zoonotic diseases, and physical risks) [68,69,70].

The presence of animals in cities can be distinguished among wild animals, food-producing animals, and companion animals as described below.

*Wild animals*: In recent times, the presence of wild animals has been investigated in the case of mammals [71] in terms of numbers and types of presence. Enlarging cities generate spaces for the adaptation of different species according to their body mass, diet breadth, reproductive timing, reproductive outputs, behavioral flexibility, and flying ability. These traits are considered filtering effects, allowing adaptation and persistence in an urban environment. In cities, the presence of food and ecological niches might facilitate mammalian adaptation in carnivores, primates, and rodents. Some of them are classified as urban visitors (those who enter cities periodically) or urban dwellers (those who establish their homes in urban settings). The elements that might influence the presence and different behaviors of animals are related both to the filtering traits of each species and their combination with the organization of cities (in terms of green areas, green corridors, food availability, waste production and management, space availability, level of human acceptance, and type of interaction). Birds as well as other small animals and insects inhabit urban settings; the presence of these animals might represent opportunities for biodiversity, nature conservation, and natural observation, but also for environmental monitoring activities [72,73].

In particular, insects go hand in hand with green re-naturalization in urban settings with their pollination activities [74]; moreover, there could also be positive relationships between re-greening urban spaces and animal presence.

Despite the presence of small animals living in or entering cities every day (birds, rats, beavers, squirrels, snakes, etc.), wild animals have always selected habitats outside of cities where they can find shelter and food [75]. Due to strong urbanization and impoverishment of the natural environment, nowadays wild animals penetrate urban borders and enter cities, even if cities can be challenging places for them. Since by living in or near cities animals can live longer—there are few predators—and access more food, urban areas have become animal habitats. The search for food sources is one of the first motivations that push wild animals to enter urban environments, as cities can provide easily accessible food sources for some animals (wild boars, foxes, deer, wolves, monkeys, etc.) [76]. Cities are noisy and chaotic places, and some animals have adapted by changing their behaviors [77,78,79] (i.e., the time of day that they are awake) to avoid people or to find more food or mates (or in the case of the COVID-19 pandemic, some animals managed to access more habitat spaces in the absence of human life [76]). To provide some examples of wild animals nowadays present in cities, the literature reports the presence of black bears in urban areas in North America and brown bears in some East European [80] cities. In the same way, in Mediterranean cities (i.e., Barcelona [81]), wild boars are frequently observed roaming within urban areas [82,83]. Aside from the possible risks in the case of dangerous animals or zoonotic diseases, wild animals in cities represent a natural resource that is part of natural environments, providing humans with the possibility of nature observation, listening to nonhuman sounds, and spaces for well-being and reflection.

*Food-producing animals*: The historical background of cities reminds us that animals have always lived with people and have played active roles in the self-sufficiency of families through their production (eggs, milk, meat, etc.), as well as providing a means for transportation. The city of Matera (Italy), as shown by Carlo Levi in his novel [84], is a clear example of human–animal daily urban interactions in the so-called Sassi houses [85], but also the cities of Glasgow and New York are mentioned in the literature about the topic [86]. Food-producing animals are still present in contemporary examples in many developing large cities in the world today [87,88,89]. With economic development and urbanization, the movement of people from the countryside to the city meant that the custom of raising courtyard animals was progressively lost, although not everywhere. Nowadays, instead, the desire for a return to nature and the emergence of new technological solutions are supporting the reintroduction of food-producing animals at the city level (with aquaponics, small domestic egg production, and goats); therefore, situations in which people breed animals in the city for production are increasingly widespread again. From this point of view, animals still represent a source of food in (some) cities and a solution to access fresh food. This is mostly part of the tradition but also relates to the migrant movements in many countries, as well as to the availability of innovative and sometimes unexpected techniques (see aquaponics, but also bees in cities) and social behaviors. From this point of view, the increasing demand for urban food generates space for fresh and nutritious food production by keeping animals in densely populated areas without environmental and health hazards [90].

*Companion animals*: In addition to the previous phenomena, in most cities, it is evident how the presence of companion animals (mainly dogs and cats, but also horses) is traditionally present and now quickly increasing.

Companion animals have always been present in cities; for example, there is archeological evidence that reports how approximately 14,000 years ago, domestic wolves, ancestors of the dog, lived in settlements with humans [91]. This is proof of the interactive process of domestication and socialization of animals through reciprocal evolutionary changes with humans. Based on this, it is not difficult to understand how companion animals have become increasingly important in the lives of people in recent decades. More recently, in the frame of increasing urbanization, animals have started to have an increasing role in terms of relationships and participation in family life. An unplanned societal phenomenon has emerged on a worldwide scale: the presence of companion animals in urban settings generates emerging influences both on individual and socio-cultural approaches. The scientific literature on the topic relates the high numbers of companion animals to public health, both for direct and indirect effects. In terms of the direct effects, for example, we found studies stating that companion animal keepers are more likely to survive a heart attack and have lower blood pressure than people without [92]. Regarding the indirect effects, authors have concluded that human–animal bonds have an impact both on people’s interactions and health: regular dog-walking, indeed, can trigger positive social interactions between strangers [93,94] and it helps maintain a good level of physical activity [94]. People experiencing homelessness are often accompanied by companion animals [95,96,97]. Moreover, Animal Assisted Interventions (AAI) have positive outcomes on elders, people with dementia, people with autism, and people identified as NEET, and they can prevent violence against women, among other benefits [98,99,100,101,102,103]. In this respect, companion animal–human interactions can be classified as a type of animal nature-based solution.

The presence of animals in cities generates diverse possibilities in terms of human–animal interactions and, sometimes, problematic interactions. At the same time, the presence of wild animals might be recognized by some humans as both harmful in terms of hygiene, zoonotic influences, and physical risks (direct attacks or possible accidents), and as potentially beneficial in terms of biodiversity and environmental monitoring elements [104,105,106,107].

As for wild animals, the presence of domestic animals (in the urban environment) may also present some issues in terms of zoonosis (in the case of vector-borne diseases). In the same way, the coexistence between wild and domesticated animals might increase environmental and health risks (see avian flu [108,109,110]) that would need to be addressed via government regulations.

Urban animals are increasingly seen as actors to be managed or protected by way of specific rules, norms, and actions as part of their citizenship. At the same time, there is a growing interest and understanding in terms of positive personal interactions both in domestic settings and in the public—social and educational—perspective regarding the potential of human–animal bonds. New projects/solutions on this topic, although mainly isolated cases, are under discussion and testing.

From a societal point of view, human–animal bonds are continuously evolving according to societal preferences and perspectives, such as the following examples:

1. Protection from the wild and protecting the wild.
2. Working with food-producing animals (in the past, in the present, in a retro innovative perspective).
3. Interacting with companion animals from both private/public perspectives.
4. Companion animal management in urban settings (organizing new spaces, managing the impact on waste production, and representing their rights).

From a philosophical perspective, two main alternatives are entering the debate: abolitionist approach vs. citizenship [111].

Also, cities’ approaches to human–animal interactions should be considered from an evolutionary perspective, ranging from a protective approach (especially in terms of urban hygiene, control, and containment) to more proactive and positive ones.

Meanwhile, today the presence of animals is growing in urban settings and scientific and societal attention still appears discontinuous and fragmentary, generating space for sectoral and sometimes specific and punctual interventions. In our view, this underestimation of human–animal bonds in cities and city planning should be rethought by opening a reflection on the subject and on the possible outcomes it can offer in terms of health and well-being for the future of cities.

## 4. The IN-HABIT Project

The European project Horizon 2020 IN-HABIT "INclusive Health and wellBeing In small and medium size ciTies” involves four European cities—Cordoba (Spain), Riga (Latvia), Lucca (Italy), and Nitra (Slovakia)—and it aims at increasing inclusive health and well-being through the mobilization of existing undervalued resources (culture, food, human–animal bonds, and environment). IN-HABIT is a 5-year project started in 2020, and it is now at the middle stage of its lifespan.

Regarding the city of Lucca, Italy, the main objective of the project is to create the first human–animal smart city in Europe, with an integrated human–animal policy able to mobilize such *resources* to increase local wellness for people who are less empowered and all citizens. The project works on different aspects of human–animal relationships to co-design innovative solutions able to give value to the presence of animals in urban settings as well as to their interactions with people. Starting from the recognition of the importance of this relationship for the well-being of citizens, in this direction, the IN-HABIT project is a pilot that aims to build an integrated policy of actions in different fields of intervention (urban planning, social and health fields, culture, economic field, tourism, etc.). The project, toward a participatory process involving the municipality, private citizens, NGOs, and the private sector, engaged local actors in a transition process aimed at translating common private visions of human–animal relationships into a public perspective and engagement around the topic.

Since the beginning of the project, special attention has been given to the active involvement of the local population and stakeholders through participatory processes, since it is important that the solutions are co-designed, co-deployed, and co-managed with and by people. Most of the effort was spent on co-creating a new vision that is able to shift the presence of animals in the city (mainly companion animals) from a private–personal link to a public affair, and from perspectives toward animal protection (mainly from NGOs dedicated to animals’ protection and rights) to a more bi-directional perspective in which nonhuman animals’ citizenship could be merged with the positive outcomes of their relationships with humans. The process facilitated the co-design of innovative solutions to be introduced in the territory of Lucca, both from an infrastructural point of view and for the soft NBSs to be implemented in the city.

In the first phase of the project, a period was dedicated to the co-design of the so-called “Animal Lines”, a path that would link the old city center (the city’s ancient walls and the under-utilized surrounding green areas) with Lucca’s suburbs and peri-urban areas. The participative process within the community assisted in gathering information, needs, and ideas about what to implement inside the areas, what materials to use to create an accessible place, and how to make the areas comfortable for both people and their companion animals. Along the path, where simple interventions will be implemented to adapt the existing cycle paths or pedestrian paths to become more companion animal-friendly (generally known by the common term “*pet-friendly*”), different areas have been built (“relational areas”) that are accessible to people and their companion animals. These spaces are aimed at fostering and facilitating human–animal relationships and, consequently, social relations and inclusion of the most fragile subjects. The areas have already been inaugurated and are open to people and their companion animals.

Subsequent to the co-design of these infrastructural interventions, another participatory phase started with a focus on possible connections between animals, people, and various urban policies such as tourism, education, social policies, policies related to the enhancement of companion animals’ related economic and professional activities, and activation of responsible citizenship. From the first discussions with the various stakeholders involved in the process, several innovative ideas emerged and provided a clear definition of the needs of the territory and a convergence of requests on the same initiatives that could, therefore, arouse integrations between the various groups of interest.

**Figure 2:**
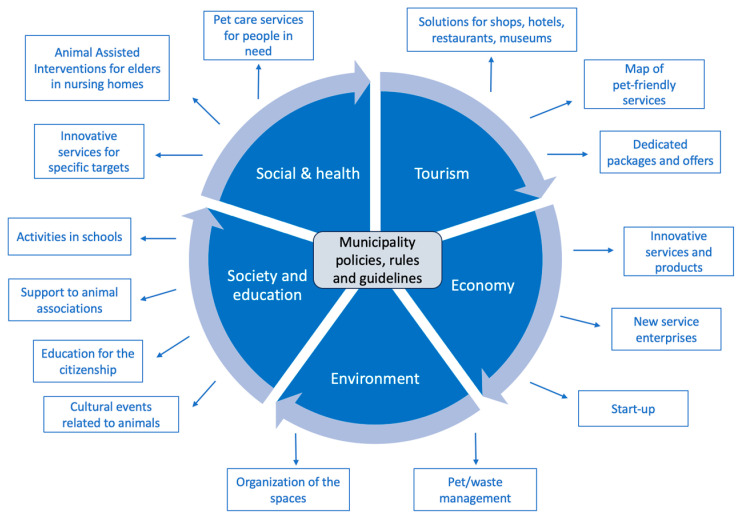
Proposed intervention areas (elaboration from the authors).

Possible intervention areas that are in the co-design and co-development phases with the Lucca partners as well as with the involved IN-HUB’s stakeholders are shown in Figure 2.

The possibility of diverse deployment of animal NBSs in the city of Lucca (and in cities in general) normally requires diverse competencies such as

- Social policies and health: AAI, innovative services for and with animals, but also the involvement of voluntary associations and the enhancement of volunteers’ attitudes toward young members of society by involving companion animals (in the facilitation of animal shelter workshops or by providing support to elders with companion animals);
- Education through activities with and in the schools: educational activities about the presence of animals in the city and interactions with citizens and cultural initiatives devoted to the links between culture and animals (from cartoons to books and from theatrical comedies to outdoor activities);
- Environment management: the organization of spaces, animal/waste management (which has an increasing impact in the project cities), and the management of wild animals and their (re)discovery in the city;
- Public building and transportation policies: to design and build suitable spaces for human–nonhuman animal interactions (i.e., Animal Lines) and or to facilitate the access of companion animals to diverse kinds of public transportation;
- Economic sector: to develop and support the (re)organization of innovative economic activities and services devoted to human–nonhuman animal interactions and to ensure the rights of companion animals (conventional and non-conventional);
- Tourism sector: to enhance the Lucca city experience as a combined one for people and their companion animals, supporting the reorganization of existing services (hotels, restaurants, museums, guided tours, supporting info, events, and games) into more “*pet-friendly*” ones;
- Municipal governance: to build a chart, a strategy plan, as well as an action plan able to integrate into one policy the set of norms, rules, procedures, and budget expenditure devoted to an integrated urban animal policy.

**Figure 3:**
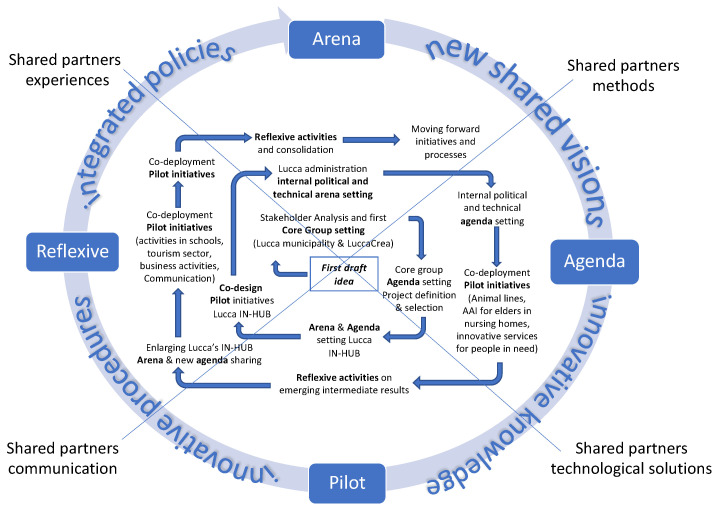
Recap of the process (elaboration from the authors).

At this moment, the project is in the middle of its lifespan, following the complete change in the Lucca political administration after the election phase in June 2022. The project was, and still is, highly demanding in terms of collective knowledge creation, vision sharing, and public–private–people integration. Moreover, the project requires the translation of the possible solutions into more integrated policies able to involve different members (technical and political) of the municipality and the integration of new paths into more ordinary processes. The full integration of political dynamics into a research project was, and still is, challenging, and from one side it is demanding in terms of political negotiation and adaptation of the project objectives, milestones, and deadlines to the requirements of political and administrative staff. At the same time, the slower the process, the deeper the opportunity to translate the research project into a real transformative initiative for the city. From this point of view, the transition process established with the IN-HUB, the agenda setting, the organization of pilots, and the ongoing reflection on the achievement as well as on the delays, are part of a collective learning activity (Figure 3). This seems to be necessary to mobilize the existing animal *resources* into animal NBSs able to increase the quality of life for all citizens from a public perspective and in the provision of public goods in requalified public spaces.

The process behind the transformation of existing animals into a public resource might be framed into different possible steps, such as

1. General idea sharing (animals as NBSs).
2. Converging vision building toward:
  - Internal communication with the administration (technical staff, political staff, and integrating sectors);
  - External communication with external stakeholders (economic sector, professionals, citizens, NGOs operating in different sectors, citizens, schools, diverse related services, i.e., dog shelters).
3. Specific co-design and co-deployment for diverse single innovative solutions.

Regarding the general process, a specific focus should be given to the overall infrastructure for a larger dialogue and communication effort. This focus should not only focus on more traditional participatory processes able to involve local private groups (NGOs, enterprises) and citizens (families, children, and youth), but it should also provide specific attention to the involvement of community building and active communication initiatives inside the municipality itself and the participation of its technical staff.

More specifically, the first attempt to reorganize public spaces to support better human–nonhuman animal interactions and to valorize animal NBSs to support local quality of life was carried out. The so-called “Animal Lines” pathway initiative was designed and rebuilt with specific spaces (i.e., relational areas) for human–dog interactions. The spaces are accessible for people with disabilities, and they can support an interactive dialogue with dogs but also with wild animals along the path. The relational areas are connected by way of the Animal Lines paths making the peri-urban areas (the natural park area on the Serchio River) more connected with the ancient Lucca medieval walls and the Roman Nottolini aqueduct on the way to Monte Pisano (Pisa side). Along the paths, people might walk, run, stop, and talk with other citizens or experience the city (in the case of tourists).

In addition to the so-called “hard solutions” (the infrastructural interventions), some soft ones have also been developed.

Regarding single solutions, a strong process of learning exchange and procedure re-design was installed to re-frame the possible valorization of human–nonhuman animal interactions in two nursing homes for elders in the city of Lucca.

An agreement with three NGO experts in AAI and the social workers of the two nursing homes was designed and weekly activities with diverse groups of elders were organized. The main outcomes are under scientific evaluation (both for animals and elders) and, in the meantime, focus groups have been organized to monitor and co-evaluate the process with the actors involved and to assess the qualitative results. In this case, different elements enter the game, such as specific knowledge, responsibility sharing among institutions and new private actors, the definition of a new commitment of NGOs and private citizens to the innovative human–animal perspective, and the opportunity to redesign roles and procedures in the provision of effective opportunities for elders’ quality of life in the nursing homes.

A second service is under co-design with other NGOs and the municipality to support the temporary needs of people regarding the management of their companion animals to reduce anxiety, as well as preventing animal abandonment in the case of temporary health limitations of an animal’s human companion (hospital recovery or temporary disabilities in the case of an isolated person).

In the next phase of the project, educational activities will be planned with primary and secondary schools for educational purposes and interaction facilities. A card game has been designed and deployed in order to gamify the educational experience.

An app engaging citizens and tourists has been designed, and it is going to be tested to engage people in missions linked to human–nonhuman animal experiences.

Meanwhile, some tourist services are under discussion with hotel managers and other economic activities (restaurants, animal shops and animal services, museums, and tour guides).

The IN-HABIT project, through participation, implementation, and new services, aims to develop the effectiveness of solutions based on a new relationship between people and animals to codify them into an integrated policy to be managed in the future and transferred to other cities interested in replicating the experience of Lucca.

At the implementation stage, the IN-HABIT project will demonstrate, at the same time, the high potential of what we can call animal (N)BSs, their abundant availability in most urban settings, and the opportunity for their mobilization in a more public-wide perspective, thus supporting the health and the quality of life of both human and nonhuman animals at the city level. 

## 5. Animals as Nature-Based Solutions and New Opportunities: The Space for Innovative Policies

The increase in the number of animals in cities is connected to evolutionary awareness/needs/demands/opportunities linked to human–nonhuman animal bonds and the responsibilities related to the human counterpart. From the economic point of view, such evidence is confirmed by the growing impact of the so-called “*pet economy*” that is increasingly affecting Western economies [112,113].

Linked to the private interests of the people, the increasing number of animals in cities and societies generates innovative opportunities in terms of societal/health impacts [114,115,116,117,118,119] and the organization of new economic opportunities linked to devoted products and services delivered. Such growing attention has clear implications in the behavior of single persons [120], inside family organizations [121], and for society [122,123].

The presence of animals (wild, food-producing, and companion animals) in the urban setting is more evident every day. However, there is a lack of reflection in the literature on the possible impact of animals on public good provision from the perspective of supportive NBSs to increase the quality of life and well-being both for human and nonhuman animals in cities. From our perspective, animal NBSs in cities might generate positive interactions with wild animals in terms of contact with nature, education about nature, education about sustainability, exploration and observation of nature, and coexistence with nature [79,124]. In such respects, specific planning initiatives can be organized at the city level (by designing specific paths, organizing cultural and educational events and activities, and involving citizens in participatory scientific activities).

Moreover, interest in food-producing animals is changing in cities. Considering the threat of future food scarcity, attention to food-producing animals might increase, although ideally based on new perspectives as part of urban agriculture initiatives. Food-producing animals might be differently involved in local urban communities, but also innovative entrepreneurial (in the case of aquaponic or small husbandry activities in cities or peri-urban areas, or rooftop gardens) or inclusive social innovation projects [125,126,127,128]. From this point of view, depending on the location (developed vs. so-called “developing countries”), on the one hand, new small productive activities are re-emerging [129]; on the other hand, more traditional ones might be reframed and improved to reduce existing hygiene risks.

Increasing attention should be dedicated to companion animals as part of the existing and growing components of nonhuman citizens (in addition to wild and food-producing animals) to consider their right to citizenship and the implication of their growing presence in urban environments. In addition to the positive (personal vs. societal) effects of companion animals in cities, there are also some problematic impacts, such as unattended dogs and dogs perceived to be aggressive or simply unfriendly [130,131], or potential risks (including bites, allergies, or biosecurity and zoonotic diseases [132,133,134,135], as well as physical injury, vehicle strikes, and property damage [136,137,138]). To shift the attention from animals as isolated/private components in the urban area to more collective nature-based solutions able to improve the everyday lives of city dwellers has some implications on urban planning and policies, as the IN-HABIT project is trying to explore in the city of Lucca. The project generates many new possible intersections at the municipal level with diverse fields of activity:

- Veterinary public health sector: the framing of new services, procedures, and rules for the management of innovative NBSs with animals, and the sanitary implications related to their new and growing presence (such as the sanitary impact of companion animal waste in terms of microbiological effects in interactions with humans [139]);
- Public building sector: the introduction of specific city plans for the presence of animals—wild (i.e., birds and insects)/food-producing (i.e., aquaponics, bees, and chickens) and companion animals—in the city, such as spaces for dedicated outdoor activities;
- Environmental sector: the management of the new wastes produced by animals—mainly companion ones—outdoors and at the home level, and reorganizing strategies in environment management (parks and green areas);
- Educational/cultural dedicated policies: policies for young people and citizens to enhance opportunities for education regarding animals that can be present in cities;
- Social and health policies: the introduction of new plans and programs able to explore the potential of human–nonhuman animal (mainly companion ones) bonds and their positive outcomes for diverse targets of urban populations with specific needs (elders, people who are less empowered, people experiencing homelessness, people identified as NEET, migrants, people with autism, people with disabilities, etc.);
- Economic activation: the support of innovative services and activities devoted to the increasing presence of animals in cities and, therefore, generating job—also innovation—opportunities for young people and all;
- Tourism sector: the exploration of the potential of the increasing number of people and families attending tourism opportunities with their companion animals (mainly dogs and cats);
- Public offices: the generation of new policies concerning the presence of animals (mainly companion ones) in public and private offices [140];
- Among others.

Looking at the scientific research on animals linked to NBSs, examples such as the study by Malhi et al. [141] were found, which explored how conservation or restoration of large wild animal populations might influence climate mitigation and the adaptation potential of ecosystems. Moreover, Berzaghi et al. [142] investigated how the incorporation of the carbon services of wild animals into financial markets has the potential to benefit both climate and conservation.

One of the few articles that directly refer to animals as NBSs is that of Danby and Grajfoner [143], in which they tried to critically analyze human–equine tourism experiences and how they can be recognized as NBSs for mutually enhancing psychological well-being. From this study, the authors could deduce how nonhumans are fundamental to increasing human well-being and mental health owing to an active mutually beneficial relationship between humans and nonhumans formed within natural spaces.

According to the IUCN classification [35], animal (N)BSs are neither green nor blue solutions (a red color could be added from this point), and they are mainly related to issues such as food security (food-producing animals) and human health. According to the EU view, animal (N)BSs can also be linked to the provision of ecosystem services related to biodiversity, especially for some specific outcomes (such as the presence of insects). From the point of view of the management process, we can consider the promotion of animal (red) NBSs to fit with the need to address some societal challenges from environmental, social, and economic perspectives. They might improve:

- Urban biodiversity (especially by considering wild animals [144,145]);
- Urban quality of life for citizens by boosting human–nonhuman animal bonds and their outcomes;
- Economic and job creation impact related to the enhancement of new devoted economic activities and initiatives (i.e., organization of wildlife watching services in collaboration with environmental guides—for wild animals—but also innovative services for the management of companion animals), as well as to the increased attractiveness of “*pet-friendly*” cities (i.e., specific services dedicated to tourists moving with companion animals, kindergartens for companion animals offered while owners are visiting museums, specific veterinary and educational services, sports activities, etc.) nowadays [146].

The Lucca project presents a new approach toward more integrated policies able to valorize animal NBSs that might be highly demanding in terms of innovative governance processes, both internally to the public institutions and at the public–private–people partnerships level. To move forward in such a direction requires progressive adaptive and evolutionary management in both public and private actions, but also the organization of more interdependent decision paths among organized citizens (in the form of citizen observatories or councils) and public decision procedures. Before that, the development of new collective knowledge—which might be built toward transition pathways until adaptive procedures and juridical rules in both the local society and the mainstream are established—is needed, as the ongoing process in the European project Horizon 2020 IN-HABIT concerning the city of Lucca (Italy) is demonstrating.

## 6. Discussion and Conclusions

NBSs are always seen and tested as suitable solutions able to enhance the quality of life for people living in urban areas, especially in the face of emerging environmental, social, and economic challenges. In our study, we observed the lack of the valorization of animals for this purpose, despite their growing presence and evidence in both our cities and our societal commitment to their citizenship. Different from other NBSs, animal NBSs are two-sided solutions. On the one hand, they give more emphasis and attention to the citizenship rights of animals in urban spaces, their possible impact, and the demand for greater attention to be paid to them and their needs in an urbanized society. On the other hand, by looking at animals as offering NBSs, there are emerging opportunities that might be explored to generate innovative and effective positive interactions with human beings and their needs. The mobilization of what we call animal NBSs demands the organization of a process of social innovation able to involve most of the public–private–people actors in transition paths to reshape common visions and actions toward the innovation of meanings about what we consider with regard to the animals living around and with us. The reshaping of public spaces from the perspective of more open interactions with animals, revising public policies and activities, and reorganizing economic and social opportunities around the valorization of human–animal bonds present opportunities for society to learn and improve, as the IN-HABIT project aims to demonstrate.

## Figures and Tables

**Table 1:** Types of challenges addressed by NBSs reported in the literature—22 empirical studies out of 40 selected publications (elaboration on the work of Ershad Sarabi et al. [38]).

NBS Challenges	Number of Papers
Water management	14
Climate change mitigation and adaptation	10
Public health and well-being	10
Social justice and social cohesion	9
Urban regeneration	8
Participatory planning and governance	8
Green space management	7
Economic opportunities and green jobs	5
Air quality	4
Coastal resilience	2

## Data Availability

Data are contained within the article.
